# Knowledge and Awareness of Hepatitis B Infection Among Young Adults in Ekiti, Nigeria: Implications for Education and Vaccination

**DOI:** 10.7759/cureus.49778

**Published:** 2023-12-01

**Authors:** Tosin A Agbesanwa, Felix O Aina, Azeez O Ibrahim

**Affiliations:** 1 Family Medicine, Ekiti State University, Ado Ekiti, NGA; 2 Family Medicine, Federal Teaching Hospital Ido Ekiti, Ido Ekiti, NGA

**Keywords:** young adult, vaccination, education, hepatitis b infection, knowledge and awareness

## Abstract

Aim

Hepatitis B is a preventable infection with transmission of the virus through sex, by blood and from mother to child during childbirth. Young adults are prone to some of these risk factors, yet data on awareness and knowledge of hepatitis B among them is still limited in Nigeria and none from our locality. The purpose of this study was to assess the knowledge and awareness of hepatitis B among young adults attending a tertiary health institution in Nigeria with the aim of giving recommendations based on the findings.

Methods

A total of 223 young adults who attended the adolescent and young adult clinic of the Family Medicine Department of the Ekiti State University Teaching Hospital, Ado Ekiti between 1 March and 31 May 2023 were analyzed using a predesigned self-administered questionnaire. Knowledge of hepatitis B was assessed by asking 12 questions relating to awareness of the disease, basic knowledge, route of transmission, the seriousness of the disease when compared to human immunodeficiency virus (HIV), complications from the disease, their vaccination status and the number of doses received by the participants.

Results

The prevalence of awareness of hepatitis B among respondents was 88 (39.5%). Of the 223 respondents studied, 158 (70.9%) had a poor knowledge score of the disease. There was a significant association between the highest educational background of the respondents and awareness of hepatitis B (p=0.05). Awareness of hepatitis B was also associated with previous participation in any health screening (p=0.04) and vaccine awareness (p=<0.001). The majority of the respondents with good knowledge scores of hepatitis B preferred social media for disseminating information about their health (p=0.03). Out of all the participants studied, only one (0.4%) had completed the three doses of the hepatitis B vaccine with 11 (4.9%) of them yet to get fully vaccinated.

Conclusion

Efforts are needed to target social media platforms with information about hepatitis B and vaccination. With awareness campaigns of hepatitis B directed at various social media platforms, this population at risk could be educated about the disease and the benefits of vaccination. The low vaccination state among the respondents brought to the fore the urgent need for the government to ensure the provision of appropriate interventions for viral hepatitis among young adults.

## Introduction

Hepatitis B virus infection is a vaccine-preventable disease [1]. The transmission occurs from exposure to infected persons through sex, by blood, and from mother to child during childbirth [2]. Hepatitis B is a major public health challenge globally often causing liver cirrhosis, cancer, and failure in infected people [3]. It has been reported that people infected with hepatitis B account for 30% and 57% of those with liver cirrhosis and cancer respectively [4,5].

This infection presents in an asymptomatic way in 50% to 70% of older children and adults [6]. Symptomatic people often present with clinical manifestations between 120 days and five years of getting infected [6]. While two-thirds (66.7%) of those infected are asymptomatic, others may proceed to icteric hepatitis and occasionally fulminant hepatic failure [5]. It has been reported that most of the 20 million people living with viral hepatitis in Nigeria are undiagnosed leading to the possibility of transmission and severe complications in the carrier [6].

Young adults are people included in the ages 15 to 24 years [7]. For this age group, it is expected that most of them would have been vaccinated as children during the expected childhood immunization against killer diseases in Nigeria. However, it is worth noting that the hepatitis B vaccine was introduced into the National Programme of Immunization (NPI) in Nigeria in 2004 [8]. The implication of this is that many of those born before 2004 who fall within the age group of young adults might not have been vaccinated. This predisposes them to being infected if they are exposed to risks of transmission like the injection of drugs and involvement in less safe sexual behavior as reported in the literature [8]. Four doses of hepatitis B vaccine are regularly given to infants born in the country with the first and the last dose given within 24 hours of birth and 14 weeks after delivery respectively [8]. The other two doses are at 6 weeks and 10 weeks post-delivery. However, there are factors that negatively affect the vaccine uptake in Nigeria as and when due. This includes children born on weekends in some areas when there are no health workers during that period to vaccinate them and some northern regions of the country where religious sentiments still negatively influence vaccine uptake generally [9,10].

Interventions have been done to stem the tide of occurrence of hepatitis B in Nigeria [10]. The 28th of July every year has been declared as World Hepatitis Day since 2010 [11]. During this period, awareness campaigns targeting the general population which are run by government agencies, non-governmental organizations, and some health professional bodies are usually taken to hospitals and other public places in Nigeria to sensitize on the dangers of hepatitis [10]. Though schools are included in some of these campaigns, no specific program has been targeted at young adults who might not pay attention at such gatherings yet engage in less safe sexual behaviors that predispose them to getting infected [12,13].

Data on awareness and knowledge of hepatitis among young adults is still limited in Nigeria and none from our locality. The aim of this study therefore was to assess the knowledge and awareness of hepatitis B among young adults attending a tertiary health institution with the view to give appropriate recommendations based on the findings.

## Materials and methods

This was a prospective cross-sectional study among young adults who attended the adolescents’ and young adult clinic of the Family Medicine department of the Ekiti State University Teaching Hospital (EKSUTH), Ado Ekiti from 1st March to 31st May 2023. Other clinics which run in the department include the general outpatient, older adult and lifestyle clinics. The adolescents’ and young adult clinic specifically attends to the health care needs of individuals who are between 18 and 24 years irrespective of their gender orientation and disease state. EKSUTH is a state government-owned hospital which serves the health care needs of over three million inhabitants (18 years and above) of the state and from the neighboring states of Osun, Ondo and Kogi. The adolescent and young adult clinic caters to the healthcare needs of young adults.

Study population

Young people aged 18 to 24 years attend the adolescent and young adult clinic of the Family Medicine Department of EKSUTH.

Sampling

All consenting young people aged 18 to 24 years who presented to the adolescents and young adult clinic of the Family Medicine Department were included in the study. The survey was done using a predesigned self-administered questionnaire which was developed from existing literatures on similar studies [8,14,15]. The questions included inquiry on the personal data of the respondents, general knowledge and awareness of hepatitis B and knowledge about the route of transmission of the disease. Face validity was done by an expert in the Family Medicine specialty. In order to ascertain the validity of the knowledge subscale, two specialist physicians in the field of infectious disease reviewed the questionnaire. To further evaluate the reliability of the questionnaire, 20 students of the College of Nursing, EKSUTH had the questionnaires administered to them on two different occasions with an interval of one month which showed moderate-to-high reproducibility. A pretext of the questionnaire was done on 50 young people attending the family medicine clinic of the Federal Teaching Hospital, Ido Ekiti to rule out any misleading or confusing questions.

Data management

The obtained data was coded and analyzed using Statistical Package for the Social Sciences (IBM SPSS Statistics for Windows, IBM Corp., Version 25.0, Armonk, NY). Analysis using descriptive statistics was performed to obtain the general characteristics of the study participants.

Statistical analysis

The general characteristics of the patients were analyzed using descriptive statistics. The categorical variables were reported as frequency distribution and proportions with 95% confidence intervals and were compared using the chi-square test or Fisher’s exact test.

Ethical considerations

Ethical clearance with protocol number EKSUTH/A67/2023/03/004 was obtained for the study from the Ethics and Research Committee of the EKSUTH before the commencement of the study. The confidentiality of patient information was protected through the non-usage of patients’ identifiers on the data collection form.

Instrument used

An adapted questionnaire (Appendix 1) was developed from existing literatures [8,14,15]. The knowledge of hepatitis B was assessed by asking 12 questions relating to awareness of the disease, basic knowledge, route of transmission, the seriousness of the disease when compared to human immunodeficiency virus (HIV), complications from the disease and vaccination status cum number of doses received by the participants. In this study, a score of less than 6 was scored poor, 7 to 9 was scored as moderate while 10 to 12 was categorized as good score.

## Results

Demographic variables of respondents studied

A total of 223 young people (33.8% males and 66.2% females) were recruited in the study. The demographic characteristics of the participants are displayed in Table 1.

**Table 1:** Demographic variables of respondents

Variables		Frequency (N = 223)	Percentage (%)
Age (Years)	18-19	51	22.9
	20-24	172	77.1
Gender	Male	74	33.8
	Female	149	66.2
Highest education attained	Primary School	16	7.2
	Secondary school	38	17
	Tertiary Institution	121	54.3
	Postgraduate	48	21.5
Total		223	100

Prevalence of awareness of hepatitis B virus among respondents studied

Eighty-eight (88) participants (39.5%) had heard about hepatitis B prior to the study as shown in Table 2.

**Table 2:** Prevalence of awareness of hepatitis B among respondents studied

Awareness of Hepatitis B	Frequency (N = 223)	Percentage (%)
Yes	88	39.5
No	135	60.5

Sociodemographic variables and awareness of hepatitis B

In Table 3, there was a statistically significant relationship between the highest educational background of the respondents and awareness of hepatitis B.

**Table 3:** Relationship between sociodemographic variables and awareness of hepatitis B among the respondents

Demographic variables	Awareness of Hepatitis B virus		X^2^	df	P value
	Yes	No			
Age Group					
18-19	16 (31.4)	35 (68.6)	1.811	1	0.118^*^
20-24	72 (41.9)	100 (58.1)			
Gender					
Male	31 (41.9)	43 58.1)	0.274	1	0.352^*^
Female	57 (38.3)	92 (61.7)			
Highest Educational Background					
Primary School	8 (50)	8 (50)	7.625	3	0.05
Secondary School	8 (21.1)	30 (78.9)			
Tertiary	49 (40.5)	72 (59.5)			
Post Graduate	23 (47.9)	25 (52.1)			

Awareness of hepatitis B, attendance of screening exercises, vaccine awareness and means of knowledge acquisition

In Table 4, awareness of hepatitis B had a significant relationship with participants’ previous participation in any health screening (P = 0.04) and vaccine awareness (P < 0.001). There was no statistically significant relationship with means of knowledge acquisition though a larger proportion of the respondents prefer social media as the preferred means of acquiring health-related knowledge.

**Table 4:** Relationship between awareness of hepatitis B virus and attendance of screening exercise, vaccine awareness and means of acquiring health knowledge

	Awareness of Hepatitis B virus		X^2^	df	P value
Attended Screening in last 2 years	No	Yes			
No	116 (85.9)	66 (75.0)	4.238	1	0.04
Yes	19 (14.1)	22 (25.0)			
Ever heard of Hepatitis B vaccine					
No	123 (91.1)	37 (42.0)	63.272	1	< 0.001
Yes	12 (8.9)	51 (58.0)			
Medium preferred to acquire knowledge					
Television	7 (5.2)	5 (5.7)	2.998	2	0.22
Social Media	86 (63.7)	46 (52.3)			
Doctor’s consultation	42 (31.1)	37 (42.0)			

Hepatitis B knowledge score

The hepatitis B knowledge scores of the respondents studied were 158 (70.9%), 50 (22.4%) and 15 (6.7%) for poor, moderate and good scores of the respondents respectively (Table 5).

**Table 5:** Relationship between the preferred medium of health information and hepatitis B knowledge score

Preferred Medium of Health Information	Hepatitis B Knowledge Score			X^2^	df	P value
	Poor score	Moderate score	Good score			
Television	10 (6.3)	0 (0)	2 (16.7)	11.168	4	0.03
Social Media	99 (62.7)	24 (48.0)	9 (60.0)			
Face-to-face Consultation with a Doctor	49 (31.0)	26 (52.0)	4 (26.7)			
Total	158	50	15			

Relationship between the preferred medium of health information and hepatitis B knowledge score. A larger proportion of respondents (60%) with good hepatitis B knowledge scores prefer social media as a means of assessing knowledge (Table 5).

Relationship between respondents studied and knowledge of complications from hepatitis B

In Table 6, out of all the respondents who were aware of hepatitis B, only approximately 44% knew that the disease could cause liver failure, cirrhosis or failure. It also shows that about 84% of those who were aware of the disease did not have any family history of liver disease prior to the study.

**Table 6:** Relationship between respondents studied and knowledge of complications from hepatitis B

	Awareness of Hepatitis B		X^2^	df	P value
Hepatitis B Can Cause Liver Cirrhosis, Cancer and Failure	No	Yes			
No	10 (7.4)	3 (3.4)	38.082	2	< 0.0001
I don’t know	113 (83.7)	46 (52.3)			
Yes	12 (8.9)	39 (44.3)			
Family History of Liver Disease					
No	82 (60.7)	74 (84.1)	14.013	2	0.001
I don’t know	48 (35.6)	12 (13.6)			
Yes	5 (3.7)	2 (2.3)			

Distribution of respondents who have received hepatitis B vaccine prior to the study

Less than 1% of the participants had completed the full vaccination regimen against hepatitis with about 5% stated without completion (Table 7).

**Table 7:** Distribution of respondents who have received hepatitis B vaccine prior to the study

Ever Received Hepatitis B Vaccine	Frequency (N)	Percent (%)
Yes (all 3 doses)	1	0.4
Yes (less than 3 doses)	11	4.9
No	188	84.3
I do not know	23	10.3

## Discussion

Hepatitis B virus is a serious disease that has lifelong consequences among those who are at risk. In this study, the prevalence of awareness of the disease was 39.5% as depicted in Table 2. Higher proportions have been reported in some other studies. In 2014, over 80% of students studied in Syria were aware of the disease yet unaware of its symptoms and lacked the knowledge of mode of transmission [3]. In a similar study done in another part of Nigeria, fair knowledge of hepatitis B despite high awareness (92.1%) was reported among the young adults studied [16]. Another study done among healthcare workers of similar age groups in India reported a 100% awareness rate of the disease with all participants knowing that blood and blood products were mode of transmission while 75% of them opined that sexual intercourse could be a mode of transmission [17]. The higher proportions reported in these studies might be attributed to the acquired knowledge of the participants studied who were either medical students, science students or healthcare workers.

About 40% of the participants in this study who were aware of the hepatitis B virus had tertiary education as their highest education attained (Table 3). This finding was lower than what was reported among undergraduate students by Joseph et al. where over 90% were aware of the disease [16]. This difference might be due to the peculiarity of participants studied by Joseph et al. who were medical and science students whose course of study could have influenced their level of awareness.

A quarter (25%) of the participants in this study who were aware of hepatitis B had previously attended health screening prior to their recruitment (Table 4). The reason for this low percentage might be connected to how the health screening attended by these respondents were planned and executed, and whether adequate information about hepatitis B was provided. It has been noted that for health screening to achieve the expected goal for the population at risk, it must be appropriately planned, funded and carried out [1,18]. In this regard, until there is a motivating push for young people to attend health screening and get educated about tests done for them, the disparity between awareness and attendance of health screening will persist.

There was a significant relationship between those who were aware of the disease and awareness of the hepatitis vaccine (p < 0.001). This might not be unconnected with the possibility of accessing both information about the disease and vaccination from the same source which could be both electronic and non-electronic. It is worth noting that a larger proportion of those who were aware of hepatitis B in this study got their information from social media when compared to doctor’s face-to-face consultation and television though this did not have any statistically significant association (Table 4).

In this study, over 70% of the respondents had poor scores in the knowledge of hepatitis B infection. This finding is in keeping with what was documented in a study on the knowledge of hepatitis B with a particular focus on Africa where it was reported that lack of knowledge of the disease was visibly identified [19]. Other studies done among young adults have reported similar poor knowledge about the disease and its mode of transmission [3,16,20]. Sixty percent of the respondents with good knowledge scores of hepatitis B in this study preferred social media as a means of communicating health issues to them when compared to face-to-face consultation with the doctor and television (Table 5). Studies done among young people have supported the importance individuals in this age group have placed on assessing health and other information via social media [21,22].

Only one respondent (0.4%) in this study had received the three doses of hepatitis B vaccine as required [5] with another 11 (4.9%) who had gotten less than the three doses (Table 7). Elegbede et al. reported a higher proportion of 26% hepatitis B vaccine uptake of at least one dose in their study [23]. This low prevalence of vaccination might be because of poor awareness and knowledge of the disease entity. It has been documented that the challenges to vaccination against hepatitis B among young people were due to poor education, cost of vaccine and patient compliance [24]. Unfortunately, there has not been an improvement from this reported finding in the last 30 years. In the United States, it has been reported that there had been a decline in vaccination of adolescents thus likely increasing the susceptibility to infection [25]. In a similar manner, a study among young adults in Nigeria has reported a little over a quarter of this age group have received one or more doses of hepatitis B vaccination [23].

## Conclusions

Efforts are needed at targeting social media platforms with information about hepatitis B and vaccination for the benefit of young adults. With awareness campaigns of hepatitis B directed at various social media platforms, this population at risk could be educated about the disease and the benefits of vaccination. In addition, the organizers of health screenings need to educate young adults about the medical tests done during the period. The low vaccination state among the respondents brought to the fore the urgent need for the government to ensure the provision of appropriate interventions for hepatitis B among young adults.

Limitations of the study

This study being a cross-sectional one presented a difficulty in drawing a predictive conclusion based on the differences observed between assessed variables. In addition to this, the findings of this study have to be generalized with caution because it is a hospital-based one.

Conflict of interest

The authors declare that there are no conflicts of interest.

Relevance of the study

This study has brought to the fore the imperative of educating this population at risk, on hepatitis B which, though a preventable disease, has the tendency of developing into serious complications when contracted.

## Appendices

Appendix 1

Questionnaire on Knowledge and Awareness of Hepatitis B Infection Among Young Adults in Ekiti, Nigeria: Implications for Education and Vaccination

(Kindly tick the option you considered most appropriate to you)

SECTION A: Sociodemographic Characteristics

1. Age  ________________                               

2. Gender   

i. Male           

ii. Female 

3. What is your highest educational attainment?

i. Primary school     

ii. Secondary School     

iii. Tertiary Institution  

iv. Postgraduate 

SECTION B: General Knowledge and Awareness of Hepatitis B

(each correct response from questions 3 to 5 a-j attracts 1 mark each)

1. Have you ever heard about Hepatitis B virus?   

i. No             

ii. Yes   

2. I have Family History of Liver disease

i. No                           

ii. I do not know                   

iii. Yes   

3. How serious do you think hepatitis B virus is compared to HIV? 

i. Less serious than HIV                             

ii. As serious as HIV        

iii. More serious than HIV                           

iv. I don’t know   

4. Hepatitis B can increase the risk of contracting liver cirrhosis, cancer, and/or failure?

i. No                           

ii. I do not know                   

iii. Yes   

Knowledge about transmission risk of Hepatitis B

5. How can someone be infected with Hepatitis B virus? (put a circle in the most appropriate option)

a. Airborne         

i. No           

ii. Yes  

b. Hereditary         

i. No           

ii. Yes  

c. Touching an infected person              

i. No         

ii. Yes  

d. Dining with infected person               

i. No           

ii. Yes  

e. Through feeding or from the toilet       

i. No           

ii. Yes  

f. Having sex with infected persons         

i. No           

ii. Yes  

g. Through contact with blood of an infected person   

i. No   

ii. Yes  

h. Using contaminated sharp objects           

i. No           

ii. Yes  

i.  Sharing of needles with other people       

i. No           

ii. Yes  

j. Mother-to-child transmission              

i. No           

ii. Yes  

Hepatitis B Vaccination Awareness and Status

1. Have you ever heard of Hepatitis B vaccine before?   

i. No   

ii. Yes  

2. Vaccination can prevent Hepatitis B infection       

i. No           

ii. Yes  

3. Have you ever received Hepatitis B vaccine before?    

i. No   

ii. Yes  

If your answer to question 2 above is ‘No’, answer question 4; if it is ‘Yes’, go to question 5

4. Why have you not received hepatitis B vaccination? (Please tick all the correct options if your answer is more than one

i. I am not aware of Hepatitis B vaccine         

ii. I do not know where to go and receive it 

iii. I don’t have time                     

iv. It is too expensive             

v. I don’t see the need                           

vi. I am afraid of contracting the virus from the vaccine  

vii. Others (Please state) ___________________________________________________

5. If your answer to question 2 is ‘Yes’, how many doses of hepatitis B vaccine have you received?

i. 1 dose               

ii. 2 doses           

iii. 3 doses          

Knowing more about Hepatitis B

1. What Information topics that you will like to know more about Hepatitis B virus

i. Disease prevention       

ii. Disease symptoms and complications 

iii. The treatment of the disease      

2. Medium through which you will prefer to have more information about Hepatitis B

i. Television         

ii. Social Media         

iii. Doctor’s face to face consultation   

Screening for Hepatitis B

I have attended health screenings in the past 2 years? 

i. No     

ii. Yes  

## References

[1] Kusi KA, van der Puije W, Asandem DA (2023). World Hepatitis day 2021 - screening and vaccination against Hepatitis B virus in Accra, Ghana. BMC Public Health.

[2] Brouard C, Gautier A, Saboni L, Jestin C, Semaille C, Beltzer N (2013). Hepatitis B knowledge, perceptions and practices in the French general population: the room for improvement. BMC Public Health.

[3] Ibrahim N, Idris A (2014). Hepatitis B awareness among medical students and their vaccination status at Syrian Private University. Hepat Res Treat.

[4] Perz JF, Armstrong GL, Farrington LA, Hutin YJ, Bell BP (2006). The contributions of hepatitis B virus and hepatitis C virus infections to cirrhosis and primary liver cancer worldwide. J Hepatol.

[5] Burns GS, Thompson AJ (2014). Viral hepatitis B: clinical and epidemiological characteristics. Cold Spring Harb Perspect Med.

[6] (2016). National Guidelines for the Prevention, Treatment and Care of Viral Hepatitis in Nigeria. https://www.hepb.org/assets/Uploads/Nigeria-Hepatitis-Guidelines-TX-guidelines.pdf.

[7] Bekker LG, Johnson L, Wallace M, Hosek S (2015). Building our youth for the future. J Int AIDS Soc.

[8] Ikobah J, Okpara H, Elemi I, Ogarepe Y, Udoh E, Ekanem E (2016). The prevalence of hepatitis B virus infection in Nigerian children prior to vaccine introduction into the National Programme on Immunization schedule. Pan Afr Med J.

[9] Bada FO, Stafford KA, Osawe S (2022). Factors associated with receipt of a timely infant birth dose of hepatitis B vaccine at a tertiary hospital in North-Central Nigeria. PLOS Glob Public Health.

[10] Alamrew Z, Bedimo M, Azage M (2013). Risky sexual practices and associated factors for HIV/AIDS infection among private college students in Bahir Dar City, Northwest Ethiopia. ISRN Public Health.

[11] Aji J, Aji MO, Ifeadike CO (2013). Adolescent sexual behaviour and practices in Nigeria: a twelve year review. Afrimedic J.

[12] (2023). In Nigeria, Boosting Viral Hepatitis Awareness and Treatment. https://www.afro.who.int/countries/nigeria/news/nigeria-boosting-viral-hepatitis-awareness-and-treatment.

[13] Ward JW, Averhoff FM, Koh HK (2011). World Hepatitis Day: a new era for hepatitis control. Lancet.

[14] Chan HL, Wong GL, Wong VW, Wong MC, Chan CY, Singh S (2022). Questionnaire survey on knowledge, attitudes, and behaviour towards viral hepatitis among the Hong Kong public. Hong Kong Med J.

[15] Eni AO, Soluade MG, Oshamika OO, Efekemo OP, Igwe TT, Onile-Ere OA (2019). Knowledge and awareness of hepatitis B virus infection in Nigeria. Ann Glob Health.

[16] Joseph AA, Joseph O, Olokoba B (2021). Knowledge of hepatitis B among young adults in a Higher Learning Institution in Nigeria and its implication on effective disease control. Microbes Infect Dis.

[17] Setia S, Gambhir R, Kapoor V, Jindal G, Garg S, Setia S (2013). Attitudes and awareness regarding hepatitis B and hepatitis C amongst health-care workers of a tertiary hospital in India. Ann Med Health Sci Res.

[18] Abesig J, Chen Y, Wang H, Sompo FM, Wu IX (2020). Prevalence of viral hepatitis B in Ghana between 2015 and 2019: a systematic review and meta-analysis. PLoS One.

[19] Mokaya J, McNaughton AL, Burbridge L (2018). A blind spot? Confronting the stigma of hepatitis B virus (HBV) infection - a systematic review. Wellcome Open Res.

[20] Ahmad A, Munn Sann L, Abdul Rahman H (2016). Factors associated with knowledge, attitude and practice related to hepatitis B and C among international students of Universiti Putra Malaysia. BMC Public Health.

[21] Hausmann JS, Touloumtzis C, White MT, Colbert JA, Gooding HC (2017). Adolescent and young adult use of social media for health and its implications. J Adolesc Health.

[22] Gong Q, Verboord M (2020). Social media use and health information seeking and sharing among young Chinese adults. J Soc Med Soc.

[23] Elegbede OE, Alabi AK, Alao TA, Sanni TA (2022). Knowledge and associated factors for the uptake of hepatitis B vaccine among nonmedical undergraduate students in a private university in Ekiti State, Nigeria. Niger J Med.

[24] Olawade DB, Wada OZ, Odetayo A, Akeju OO, Asaolu FT, Owojori GO (2022). COVID-19 vaccine hesitancy among Nigerian youths: case study of students in Southwestern Nigeria. J Educ Health Promot.

[25] Le MH, Yeo YH, So S, Gane E, Cheung RC, Nguyen MH (2020). Prevalence of hepatitis B vaccination coverage and serologic evidence of immunity among us-born children and adolescents from 1999 to 2016. JAMA Netw Open.

